# Update on Novel Targeted Therapy for Pleural Organization and Fibrosis

**DOI:** 10.3390/ijms23031587

**Published:** 2022-01-29

**Authors:** Torry A. Tucker, Steven Idell

**Affiliations:** The Department of Cellular and Molecular Biology, The University of Texas Health Science Center at Tyler, 11937 US Hwy 271, Tyler, TX 75708, USA; steven.idell@uthct.edu

**Keywords:** pleural injury, organization and scarification, pleural mesothelial cells, mesomesenchymal transition (MesoMT), intrapleural fibrinolytic therapy (IPFT), fibrinous neomatrices, pleural loculation and fibrothorax

## Abstract

Pleural injury and subsequent loculation is characterized by acute injury, sustained inflammation and, when severe, pathologic tissue reorganization. While fibrin deposition is a normal part of the injury response, disordered fibrin turnover can promote pleural loculation and, when unresolved, fibrosis of the affected area. Within this review, we present a brief discussion of the current IPFT therapies, including scuPA, for the treatment of pathologic fibrin deposition and empyema. We also discuss endogenously expressed PAI-1 and how it may affect the efficacy of IPFT therapies. We further delineate the role of pleural mesothelial cells in the progression of pleural injury and subsequent pleural remodeling resulting from matrix deposition. We also describe how pleural mesothelial cells promote pleural fibrosis as myofibroblasts via mesomesenchymal transition. Finally, we discuss novel therapeutic targets which focus on blocking and/or reversing the myofibroblast differentiation of pleural mesothelial cells for the treatment of pleural fibrosis.

## 1. Introduction

Pleural injury can develop as a result of underlying infection, collagen vascular disease or other insults and when severe can progress to local organization and fibrosis [1]. The presence of high-grade inflammatory pleural effusions or exudates, whether infectious or sterile [2,3], can promote accelerated formation of fibrin-rich pleural collections known as pleural loculations. Organization of the pleural neomatrix can ultimately result in pleural thickening or fibrothorax, with associated restrictive lung disease and limitation of lung capacity. Low pH and low glucose-containing pleural effusions or those with 3-fold elevations of the lactate dehydrogenase or evidence of infection in relatively larger pleural effusions are commonly taken as indicators of the need for invasive means of drainage [1,4,5]. Tube thoracostomy can expedite pleural drainage but when ineffective, intrapleural fibrinolytic therapy (IPFT), video-assisted thoracostomy or thoracotomy may be necessary. The invasiveness and morbidity of surgical drainage and inconsistent efficacy of IPFT in adults [1] underscore the need for better tolerated, more efficacious therapy that can prevent organizing pleural injury from eventuating in clinically important restrictive lung disease.

## 2. Organizing Pleuritis and Its Place as an Important Problem in Pleural Medicine

Pleural effusions with failed or insufficient drainage and persistent pleural loculation are commonly seen in pulmonary practice, with overall associated costs of care that may reach billions of dollars [1]. Empyema infection of the pleural space can complicate an underlying pneumonia, with florid formation of pleural loculated collections capable of impairing pleural drainage [1]. Failed pleural drainage can extend hospital stays, and mortality can approach 20 percent contingent on underlying comorbidities [1]. Pharmacotherapy is desirable to avoid the exposure of patients to surgical remedies. Unfortunately, the current mainstay of pharmacotherapy for organizing pleural injury; IPFT, is variably effective in adults [1], offering the predicate for testing of new agents in clinical trials at the time of this writing [6,7]. Contributing factors to variable outcomes of IPFT in adults include empiricism surrounding which agent or agents are best, what dose should be used for most effective and safe outcomes and what dosing schedules best achieve desired results with prompt pleural drainage.

## 3. The Pathogenesis of Pleural Organization and the Role of Aberrant Fibrin Turnover and Mesomesenchymal Transition (MesoMT)

Pleural organization that leads to disease is commonly seen as a result of severe pleural inflammation coupled with exudative pleural effusions [8]. Inflammation associated with pleural injury promotes disordered fibrin turnover. Enhanced vascular permeability resulting from this inflammation causes fibrinogen-rich plasma-like fluid to leak into the inflamed pleural space. This fibrinogen is then converted to fibrin by the local expression of soluble and cell-associated tissue factor (TF) and subsequent activation of the extrinsic coagulation pathway. Sources of TF include resident cells found within the pleural space including macrophages, mesothelial cells, fibroblasts and endothelial cells [1]. Insoluble intrapleural fibrinous collections can be degraded by plasmin, the serine protease fibrinolysin derived from plasminogen found in the plasma. Plasminogen is converted to its active form by urokinase (u) and tissue type (t) plasminogen activators (PA). uPA is also found in the plasma as the proenzyme single chain uPA. tPA is expressed by resident mesothelial cells. While both Pas are present in the injury milieu, their activities are regulated by the plasminogen activator inhibitor (PAI)-1. TNF-α and VEGF are among the many proinflammatory cytokines found in the injured pleural space [1,9,10]. In addition to recruiting inflammatory cells to the injury site, factors such as TNFα also induce the local expression of PAI-1, which irreversibly inhibits PA activity due to either tPA or uPA. The local robust elaboration of PAI-1 overwhelms endogenous PA activity and promotes florid fibrin deposition, driving pleural fibrosis (PF).

## 4. PAI-1

PAI-1 expression is reported to be increased in diverse forms of pleural injury, including empyema, malignancy, and tuberculosis [6,7,11,12,13]. Karandashova et al. showed that enhanced PAI-1 expression in the pleural space worsened injury outcomes in a tetracycline rabbit model of pleural injury [14]. In this model, injury in the context of enhanced PAI-1 expression was characterized by increased florid fibrin deposition, which promoted a close facsimile of loculation as occurs in organizing human pleural injury. Similar results were observed in a carbon black/bleomycin mouse injury model. PAI-1 overexpressing transgenic mice demonstrated worse injury than control mice [15]. Conversely, the total absence of PAI-1 in this model yielded paradoxical results. As anticipated, fibrin deposition was notably reduced in the absence of PAI-1. However, pleural remodeling, as indicated by thickening of the visceral pleura, was worsened and associated with large, neutrophilic pleural effusions. These findings suggest a secondary role for PAI-1 that involves regulation of local inflammation in pleural injury and repair.

Understanding the role of PAI-1 in extravascular fibrin deposition in the injured pleural space is critical for the development and/or refinement of treatment for fibrothorax and restrictive lung disease. In addition, therapies which directly or indirectly target this molecule represent an appealing treatment option for the treatment of pleural organization with loculation. The most common treatment for failed pleural drainage involves the intrapleural administration of exogenous Pas (IPFT), ideally in excess of the neutralizing concentrations of PAI-1. While the use of fibrinolysins has widely been accepted as therapeutic for the drainage of complicated parapneumonic effusions (Table 1), the First Multicenter Intrapleural Sepsis Trial (MIST1) using streptokinase found no significant benefit [16]. Conversely, the second MIST trial (MIST2) identified tPA in combination with DNAse [17] as being effective for the treatment of complicated parapneumonic effusions. However, these studies were limited in the scope of the dosing range and schedule and were thus empiric as they evaluated a single dose of fibrinolysin. In addition, patients in MIST2 with free-flowing and loculated pleural effusions were enrolled, which could have affected outcomes. Recently, single chain uPA was evaluated in a Phase 1 Trial (ANZCT ID: ACTRN12616001442493) [7]. This was the first dose escalation study to assess fibrinolysin efficacy in the treatment of parapneumonic effusions. This study also showed that the fibrinolytic activity of scuPA was durable in infectious pleural injury as it evaded inhibition by PAI-1 via complex with α2 macroglobulin. In this trial, the use of scuPA in doses from 50,000 to 800,000 IU was found to be safe when delivered by tube thoracostomy in patients with pleural infection, loculation and failed pleural drainage. These findings enabled the conduct of a phase 2 trial of scuPA in patients with empyema and failed pleural drainage, which is currently underway (ClinicalTrials.gov: NCT04159831). These studies also suggest that PAI-1 may represent a previously unrecognized target for the treatment of pleural loculation.

## 5. Mesothelial Cells

Dvorak and colleagues reported that fibrin is a key component of the provisional neomatrix that provides proangiogenic signals which promote vascularization [18]. As the transitional fibrin matrix is remodeled by endogenous fibrinolytic activity, it may subsequently be replaced by more persistent matrix proteins collagens 1 and 4. Transforming growth factor (TGF)-β is an anti-inflammatory and immunosuppressive cytokine that is critically important in tissue repair [19,20]. TGF-β is also profibrotic and increased in the pleural effusions of patients with infectious conditions [19,20,21]. TGF-β has three isoforms, 1-3, and is associated with a latent TGF-β binding protein. All three TGF-β isoforms are reported to induce PF [22] in various animal models. TGF-β is also a potent inducer of collagen and PAI-1, thereby contributing to disordered fibrin turnover and collagen deposition. Pleural mesothelial cells are reported to play an active role in both the initial deposition of fibrin initiated by TF and its subsequent replacement by collagen [1,23]. Pleural mesothelial cells are also reported to be a significant source of collagen in the injured pleural space [15,24,25,26].

## 6. MesoMT

TGF-β is also a potent inducer of myofibroblast differentiation in diverse cell types, including lung fibroblasts and pleural mesothelial cells [24,25,27,28,29,30,31]. Myofibroblasts appear as pleural or organizing injury in other tissues progresses. In normalcy, as tissue regeneration and repair occur, the myofibroblast population recedes. However, in chronic, unresolved injury, these myofibroblasts persist, resist apoptosis, and their population expands and directly contribute to progressive fibrosis of the affected tissue and/or organ [32]. Myofibroblast differentiation is characterized by the loss of epithelial cell markers and the acquisition of profibrotic markers including α-smooth muscle actin (SMA) [32,33]. Myofibroblast also secretes extracellular proteins including collagens and fibronectin, which can contribute to pathologic tissue reorganization, such as pleural thickening. Pleural mesothelial cell (PMC)-derived myofibroblasts via MesoMT likewise express α-SMA, collagen and fibronectin [34,35,36] and lose the expression of tight junction proteins such as E-cadherin and zona occludins 1 [36,37].

**Figure 1:**
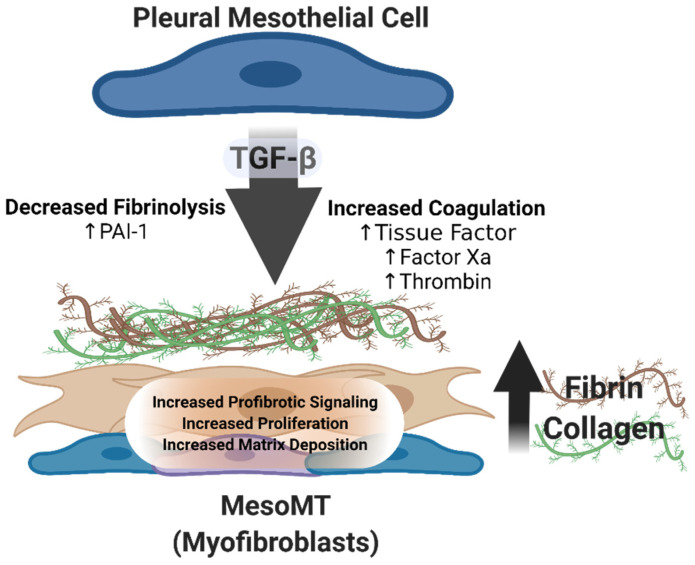
Induction of MesoMT and Extracellular Matrix Deposition. In response to local injury, the pleural space demonstrates enhanced fibrin deposition via increased local procoagulant activity resulting from increased tissue factor, Factor Xa and thrombin and decreased fibrinolysis via increased PAI-1 expression [1,12,42]. As injury progresses, increases in TGF-β and other profibrogenic mediators promote pleural mesothelial cell transition to a profibrotic phenotype (myofibroblasts) via mesomesenchymal transition (MesoMT) [25,27,28,29,37,43]. These newly transitioned myofibroblasts expand due to increased profibrotic signaling, proliferation and resistance to apoptosis. Mesothelial cell-derived myofibroblast promote pleural thickening via enhanced expression of extracellular matrix proteins including collagen and fibronectin. TGF-β and other mediators of MesoMT activate PI3K/Akt, NFκB and GSK-3β prosurvival signaling pathways. These pathways have also been shown to be critical for the induction of MesoMT [37,43]. The therapeutic targeting of these and other pathways represent the most promising targets for the treatment of PF. Created using BioRender.

Myofibroblasts are present in diverse forms of lung injury, including idiopathic pulmonary fibrosis (IPF) and PF [15,32]. How long and why these cells persist in tissue fibrosis has not been fully elucidated. Furthermore, the source and/or origin of these myofibroblasts remains unclear. In IPF, resident fibroblasts are believed to be activated to myofibroblasts, while myofibroblasts of myeloid origin may also contribute to this population [38,39]. Lama et al. further reported that a significant percentage of collagen expressing cells, presumed to be myofibroblasts, also express macrophage markers in the injured lung [39]. These myofibroblasts then become a principal driver of the disease. PF is likewise characterized by extensive expansion of the myofibroblast population, contributing to pleural rind formation. We have shown that pleural mesothelial cells directly contribute to this myofibroblast population in nonspecific human pleuritis [15]. Furthermore, the myofibroblast population has been shown to locally expand throughout injury in various preclinical models of pleural injury [15,36,37,40,41] (Figure 1).

There are multiple models of pleural injury that progress to PF, as characterized by pleural thickening, the presence of myofibroblasts and aberrant collagen deposition. In the carbon black/bleomycin pleural injury model, we and others have shown that α-SMA expressing myofibroblasts appear early in injury, 7 day [15,44]. This population progressively expands up to 21 day post initiation of injury. In this model, collagen deposition did not become apparent until 14 day and increased throughout the 21-day course. The injury did not appear to resolve over the 21 day period. In this model, greater than 90% of the α-SMA expressing myofibroblasts were mesothelial in origin [15]. Similar results were seen in the clinically relevant *S. pneumonaie* model of pleural injury [24,27,28,30,43,45]. However, in this model, significant changes in pleural thickening were apparent 7 days post initiation of injury and persisted up to 14 day post injury. Collagen deposition and α-SMA expression also occurred by 7 day. Significant decrements in lung function and volumes were likewise observed in this model and demonstrable by 7 day [25,27,28,43,45]. The TGF-β adenoviral model is also an accepted model of pleural fibrosis. In this noninjury model, ectopic expression of TGF-β, via adenoviral transduction, promotes quantifiable pleural fibrosis by 7 day [29,41], which can persist up to 64 day [41]. This model likewise demonstrated significant decrements in lung function and increased α-SMA expression in the thickened pleural rind [29]. In all of these models, impaired lung function is attributed to the resulting collagen deposition and pleural thickening, which is apparent in all three models. There are other models of pleural injury, such as the carrageenan pleurisy model [46,47]. However, this model is typically studied acutely; around 4 h after induction of injury. As such, it has not been evaluated for its ability to induce pleural fibrosis.

While TGF-β is known to induce the myofibroblast differentiation of diverse cell types including PMCs, other mediators including factor Xa, thrombin, activated protein C, plasmin and uPA are also known to induce MesoMT [15,30]. TGF-β induces mesenchymal transition of PMCs via activation of the canonical TGF-β/SMAD signaling pathway and noncanonical pathways [28,36,37,43,48]. Specifically, TGF-β has been shown to activate SMADs 2, 3 and 4 in PMCs [28,36]. Furthermore, Smads 2 and 3 were found to be critical for TGF-β mediated MesoMT [28,36]. However, the role of the inhibitory SMADs, such as SMAD7, in PMCs, if any, is unknown. TGF-β mediated MesoMT has also been reported to be dependent on activation of the non-canonical TGF-β signaling pathways, including PI3K/Akt and NFκB [37]. These pathways have been reported to be activated by other mediators of MesoMT, such as thrombin and plasmin in PMCs. Inhibition of these pathways with small molecule inhibitors or via expression of dominant negative moieties blocked induction of MesoMT with an array of MesoMT mediators [37].

## 7. Novel Targets for Interventional Therapy

While established classic TGF-β pathways are functional in MesoMT, several novel targets have been identified in preclinical studies (Table 2). TGF-β alone can induce lung fibrosis through binding to its receptor, TGFβR1. TGF-β can also regulate the expression and function of other surface receptors which represent potential therapeutic targets for the treatment of PF. NADPH oxidase 4 (NOX4) expression is notably enhanced by TGF-β and has previously been identified as a critical mediator of myofibroblast differentiation in diverse cell types, including lung fibroblasts [33,49,50,51,52,53]. While several therapeutics including GKT137831 [33] have been developed targeting NOX4 activity, the role of NOX4 and other NOXs had not been investigated in HPMCs, and few studies have been conducted to address its role in organizing pleural injury.

Qin et al. showed that NOX1 and NOX4 expression was enhanced in the pleural mesothelium of human pleuritis lung tissues compared to normal lung tissues [27]. In in vitro studies, NOX4 was enhanced by TGF-β but unaffected by other mediators of MesoMT. Conversely, NOX1 expression was enhanced by FXa and thrombin but unaffected by TGF-β. NOX-1 downregulation with targeting siRNA blocked Xa, thrombin and TGF-β mediated MesoMT. The NOX1 inhibitor ML171 could block the induction of MesoMT by thrombin but could not reverse the established MesoMT, as we have shown with other potential therapeutics [31,37,43]. In addition, NOX1 and NOX4 deficiency slowed the progression of PF in the *S. pneumonaie* model [27]. These studies provide rationale for the continued study of the NOX family as therapeutic targets for the treatment of pleural injury.

Regulators of the coagulation pathway also represent therapeutic targets for the treatment of PF. The endothelial protein C receptor (EPCR) is a recently identified target. EPCR is a receptor for activated protein C and factor VIIa, modulators of coagulation. As its name implies, the expression of EPCR was thought to be limited to endothelial cells. In this recent study, they found that EPCR is robustly expressed on the pleural surface of human lung tissues [30]. They also showed that EPCR deficiency reduced the progression of PF, as decrements in lung function and lung volume were improved in DOCK2 knockout mice. While pleural thickening was present in WT, EPCR knockout and EPCR overexpressors, EPCR knockout mice demonstrated significantly less pleural thickening than the WT and EPCR overexpressors. In addition, markers of MesoMT, including collagen and α-SMA were likewise reduced in knockout mice compared to WT controls. Inflammation was also reduced in EPCR knockout mice. Conversely, EPCR overexpression worsened injury outcomes and the expression of MesoMT markers compared to similarly treated WT mice [30]. These studies support the potential to target EPCR to advantage in PF as well as further investigation of the role of EPCR and other regulators of coagulation in the progression of PF.

uPAR also represents an attractive target for the treatment of PF. uPAR has been shown to be critical for the acquisition of aggressive cellular phenotypes in diverse cancers, including mesothelioma [54,55,56,57,58,59]. We previously showed that uPAR expression correlated with the increased aggressiveness of malignant pleural mesothelioma (MPM) lines in our ectopic model of mesothelioma [59]. Furthermore, a relatively nonaggressive MPM line with low levels of uPAR became highly aggressive and invasive when uPAR expression was enhanced [59]. Conversely, the most aggressive MPM line with the highest uPAR expression became significantly less aggressive when uPAR expression was reduced. We later reported that proinflammatory mediators could potentiate uPA enzymatic activity on PMCs by increasing uPAR RNA transcription and stabilizing uPAR surface expression [26]. We found that TNF-α and IL-1β reduced the expression of the endocytic receptor low-density lipoprotein receptor-related protein 1 (LRP1) in PMCs, increasing the half-life of uPAR at the cell surface, increasing uPA enzymatic durability, and increasing HPMC migration. These data suggest that uPAR and regulators of its activity can promote more aggressive phenotypes of PMCs.

In pleural fibrosis, uPAR expression was found to be modestly enhanced in the pleural mesothelium of pleuritis lung tissues [25]. While TGF-β did not induce uPAR expression in vitro, it robustly induced the expression of its cognate ligand uPA by PMCs. uPA binds to uPAR on the cell surface, which concentrates uPA enzymatic activity. uPA binding to uPAR also mediates signaling and causes rapid internalization and degradation of the uPA/uPAR complex [26,59]. Downregulation of either uPAR or uPA blocked the induction of TGF-β mediated MesoMT. Furthermore, uPAR mediated MesoMT was found to be uPA dependent. Because uPAR does not have a cytoplasmic domain, any internalization and/or signaling mediated by uPAR must occur through a secondary surface receptor, in this case LRP1. Accordingly, LRP1 knockdown blocked TGF-β mediated MesoMT. These findings were confirmed in our preclinical pleural infectious injury model, as uPAR deficiency protected mice against *S. pneumonaie-*mediated decrements in pleural fibrosis, lung function and volume.

Glycogen synthase kinase (GSK)-3β represents another druggable target for the treatment of pleural fibrosis. GSK-3β is a serine/threonine kinase responsible for the regulation of glycogen synthase [60,61]. GSK-3β has also been reported to be upregulated in numerous solid neoplasms, including glioblastoma [62]. We found that GSK-3β was enhanced in the pleura of lung tissues from patients with pleuritis [43]. Furthermore, down-regulation of GSK-3β in HPMCs blocked induction of MesoMT. GSK-3β has numerous phosphorylation sites that are reported to regulate its activity [60,61,63,64]. Phosphorylation of GSK-3β at Serine 9 is reported to inhibit its activity by competing with substrates for its active site. However, this inhibition can be overcome with increasing amounts of substrate. Conversely, the phosphorylation of tyrosine 216 is reported to potentiate GSK-3β activity [31,43,65,66]. In these studies, the GSK inhibitor 9-ING-41 blocked phosphorylation of the tyrosine 216, which blocked induction of MesoMT in vitro with diverse mediators. Furthermore, 9-ING-41 could reverse established MesoMT. 9-ING-41 also reduced indices of injury in the *Streptococcus pneumoniae* model of pleural injury [43], as decrements in lung function and volume were improved with 9-ING-41 treatment. Collagen and α-SMA were likewise reduced in 9-ING-41 treated mice. These effects were not limited to PF, as similar results were observed in normal and IPF lung fibroblasts. 9-ING-41 blocked TGF-β mediated fibroblast-myofibroblast differentiation. GSK-3β inhibition likewise reversed the progression of pulmonary fibrosis in a preclinical model of pulmonary fibrosis [31]. These studies strongly suggest that the therapeutic targeting of GSK-3β may have broad applications extending beyond PF to pulmonary parenchymal injury with progressive fibrosis.

The myocardin related transcription factor (MRTF) family includes members myocardin, MRTF-A and MRTF-B [67,68]. This family is reported to work in concert with the serum response factor (SRF) family to control the transcription of smooth muscle proteins. Myocardin specifically is smooth muscle cell specific and controls the expression of contractile and cytoskeletal proteins [68]. MRTF-A and -B had previously been shown to play a role in the myofibroblast differentiation of lung fibroblast and in pulmonary fibrosis [67,69,70], however the role of myocardin in myofibroblast differentiation was unknown. In this study, we found that myocardin was highly expressed in the pleura of human pleuritis tissues and that it colocalized with α-SMA. They also found that TGF-β and thrombin robustly induced myocardin and α-SMA expression in HPMCs. Myocardin knockdown blocked TGF-β, and thrombin mediated MesoMT. Conditional knockout of myocardin in the pleural mesothelial cells protected mice against TGF-β adenovirus mediated pleural fibrosis [29]. Specifically, α-SMA, myocardin and the myocardin effector calponin were reduced in the conditional myocardin deficient mice. These studies showed for the first time that myocardin played a direct role in the acquisition of profibrotic phenotype. Furthermore, these studies justified continued study of myocardin and related proteins, such as calponin, in the progression of MesoMT and pleural fibrosis.

Dedicator of cytokinesis (DOCK2) has an established role in numerous immune functions [71]. It is known to regulate lymphocyte migration and T cell activation [71]. However, until recently its role in myofibroblast differentiation was unknown. Recent work by Qian et al. found that DOCK2 was upregulated in human pleural injury [28]. They also found that DOCK2 expression was enhanced in three mouse models of pleural fibrosis. Conversely, DOCK2 deficiency blunted the progression of pleural fibrosis in a preclinical mode of PF. Specifically, impaired lung function and lung volume related to *S. pneumoniae* injury were blocked in DOCK2 deficient mice. Pleural thickening and α-SMA expression were likewise reduced in DOCK2 knockout mice. Although this study identified a role for SMAD3 and Snail in DOCK2 mediated MesoMT, other potential mechanisms require further study. For instance, DOCK2 is known to regulate GEF activity and to subsequently regulate Rac, but the role of this mechanism, if any, in MesoMT is unclear [68]. DOCK2 is also known to regulate myocardin/SRF [68,71] activity, which was shown to regulate MesoMT [29]. The contribution of these mechanisms has not been investigated in PF. However, these findings provide a strong rationale for further investigation of the role of study of DOCK2 in the pathogenesis of PF.

αβ crystallin was also identified as a therapeutic target for the treatment of both pulmonary fibrosis and pleural fibrosis. αβ crystallin is a small heat shock protein that is expressed in diverse tissues. It can serve as a chaperone, and its expression is regulated by a wide range of stressors [72]. Bellaye et al. found that αβ crystallin was upregulated in the fibrotic lesions of IPF lungs, as well as in preclinical models of pulmonary fibrosis [73]. Downregulation of αβ crystallin blocked myofibroblast differentiation by preventing TGF-β mediated SMAD4 localization to the nucleus. Conversely, overexpression of αβ crystallin increased SMAD4 localization to the nucleus, thus promoting differentiation. Aβ crystallin deficiency also blunted the progression of pulmonary fibrosis in three models of pulmonary fibrosis [73]. Aβ crystallin was also found to be increased in the pleura of fibrotic lungs. In addition, αβ crystallin deficient mice demonstrated reduced pleura thickening and collagen deposition compared to WT mice, when treated with TGF-β adenovirus [74]. SMAD4 localization was likewise negatively impacted by αβ crystallin knockdown in mesothelial cell. Predicated on these findings, targeting of αβ crystallin for the treatment of pleural fibrosis bears further study.

There are several potential therapeutic targets that have been investigated in other forms of lung fibrosis that merit investigation in PF. IPF is characterized by the extensive loss of the epithelial cells and expansion of αSMA expression myofibroblasts. Work by Marudamuthu et al. found that the caveolin spanning peptide (CSP) could inhibit airway epithelial cell apoptosis and fibroblast myoblast differentiation and expansion in preclinical models of murine pulmonary fibrosis [75,76]. They further identified a seven-peptide fragment of the CSP, CSP7, that was equally effective in three preclinical murine models of pulmonary fibrosis. The exceptional success of CSP7 in pulmonary fibrosis provides the rationale for future evaluation of its ability to mitigate PF.

Other targets that warrant further investigation in MesoMT include the mechanistic (or mammalian) target of rapamycin (mTOR). mTOR is a highly conserved serine/threonine kinase which regulates cellular growth, metabolism and survival [77,78,79,80]. The mTOR complex encompasses two pathways: complex 1, known by its association with Raptor and complex 2, which associates with Rictor. Woodcock et al. showed that the mTORC1 pathway, which regulates the eIF4F/4EBP1 translation axis, is a critical signaling node which controls collagen synthesis in human lung fibroblasts [81]. The mTORC2 pathway, which is known to potentiate AKT signaling, also activates the serum/glucocorticoid regulated kinase (SGK1). However, the role of the mTORC2 axis has yet to be fully evaluated in either IPF or PF. The therapeutic targeting of one or both of these pathways could represent effective treatment modalities for PF.

**Table 2:** Novel targets for interventional therapy.

Novel Targets	Description
GSK-3β; Glycogen Synthase Kinase 3β	A serine/threonine kinase reported to regulate the function of glycogen synthase. Found to be activated by TGF-β and other MesoMT mediators by phosphoyratlion of tyrosine 216. Inhibition of GSK-3β with 9-ING-41 reversed pleural fibrosis [31,43].
DOCK2; dedicator of cytokinesis 2	Rac1 activating protein previously shown to regulate cellular phenotype. Recently found to be upregulated in pleural injury. DOCK2 deficiency protects against *S. pneumoniae* mediated pleural fibrosis [28].
Myocardin	Smooth and cardiac muscle cell specific transcriptional coactivator of serum response factor. Found to be upregulated in pleural fibrosis and contributes to disease progression [29].
EPCR; endothelial protein C receptor	An important regulator of Protein C whose expression was thought to be limited to the endothelium. Recently found to be important in the progression of pleural fibrosis [30].
NOX1; NADPH oxidase 1	A member of the NOX family whose expression is enhanced by factor Xa and thrombin in PMCs. Recently reported to be important for the progression of pleural fibrosis in vivo [27].
uPAR; urokinase plasminogen activator receptor	A cell surface glycoprotein responsible for binding and localizing uPA to the surface of PMCs. Reported to be critical for the induction of MesoMT and the progression of PF [25,26,59].
αβ crystallin	A small heat shock protein known to be enhanced by TGF-β. Reported to promote the progression of pulmonary and pleural fibrosis [73,74].
CSP7; caveolin spanning peptide seven amino acid deletion fragment	A fragment of the caveolin spanning peptide. Reported to attenuate the progression of pulmonary fibrosis in murine models [75,76].
mTOR; mechanistic target of rapamycin	A highly conserved serine/threonine kinase which regulates diverse cellular activities. Therapeutic targeting of the mTORC1 pathway was shown to block collagen synthesis in lung fibroblasts [81].

## 8. Conclusions

The pathogenesis of pleural organization remains a complex interplay of disordered fibrin turnover, cell differentiation and subsequent pleural remodeling. Most currently available pharmacotherapies center on different forms of IPFT, which are focused on treating the immediate concern, pleural loculation that impairs pleural drainage. However, this treatment modality does not address the complexity of the underlying pathological tissue organization that occurs in organizing pleural injury. The novel targets presented here represent the most promising candidates for continued study. The addition of such novel interventions to the therapeutic armamentarium for PF may ultimately offer better approaches to improve clinical outcomes. Further investigation and clinical trial testing of the most promising candidates will be required to identify new and clinically tractable options for the treatment of PF.

## Figures and Tables

**Table 1:** Currently used fibrinolysins in the United States.

Fibrinolysin		
tPA (Alteplase)		
tPA/DNAse		
scuPA *		

* Currently in clinical trial testing and not commercially available.

## Data Availability

Not applicable.
